# An organometallic analogue of combretastatin A-4 and its apoptosis-inducing effects on lymphoma, leukemia and other tumor cells *in vitro*†

**DOI:** 10.1039/d2md00144f

**Published:** 2022-06-30

**Authors:** Liliane Abodo Onambele, Natalie Hoffmann, Lisa Kater, Lars Hemmersbach, Jörg-Martin Neudörfl, Nikolay Sitnikov, Benjamin Kater, Corazon Frias, Hans-Günther Schmalz, Aram Prokop

**Affiliations:** Department of Pediatric Oncology/Hematology, Children's Hospital of the City of Cologne Amsterdamer Str. 59 50735 Cologne Germany; Department of Pediatric Oncology/Hematology, University Medical Center Charité Campus Virchow, Augustenburger Pl. 1 13353 Berlin Germany; Department of Chemistry, University of Cologne Greinstrasse 4 50939 Cologne Germany schmalz@uni-koeln.de; Department of Pediatric Hematology/Oncology, Helios Clinic Schwerin 19055 Schwerin Germany Aram.Prokop@helios-gesundheit.de; MSH Medical School Hamburg Am Kaiserkai 1 20457 Hamburg Germany

## Abstract

Hexacarbonyl[1,3-dimethoxy-5-((4′-methoxyphenyl)ethynyl)benzene]dicobalt (NAHO27), an organometallic analogue of combretastatin A-4, has been synthesized and its activity against lymphoma, leukemia, breast cancer and melanoma cells has been investigated. It was shown that NAHO27 specifically induces apoptosis in BJAB lymphoma and Nalm-6 leukemia cells at low micromolar concentration and does not affect normal leukocytes *in vitro*. It also proved to be active against vincristine and daunorubicin resistant leukemia cell lines with p-glycoprotein-caused multidrug resistance and showed a pronounced (550%) synergistic effect when co-applied with vincristine at very low concentrations. Mechanistic investigations revealed NAHO27 to induce apoptosis *via* the mitochondrial (intrinsic) pathway as reflected by the processing of caspases 3 and 9, the involvement of Bcl-2 and smac/DIABLO, and the reduction of mitochondrial membrane potential. Gene expression analysis and protein expression analysis *via* western blot showed an upregulation of the proapoptotic protein harakiri by 9%.

## Abstract 2

The diarylethyne cobalt carbonyl complex NAHO27 selectively induces apoptosis in cancer cells at low μM concentrations *via* the mitochondrial pathway. It is also active against resistant cells, showing a strong synergistic effect with vincristine.

## Graphical abstract

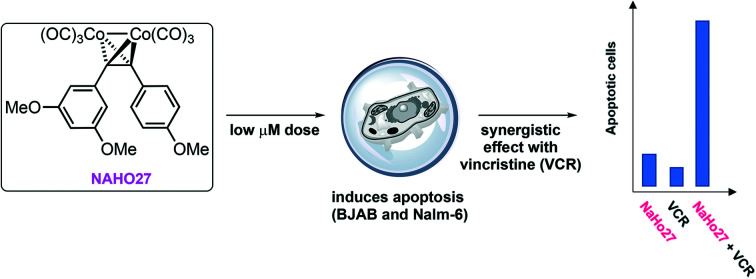

## Introduction

Cancer is one of the most dreaded diseases around the world. Almost 17 million cases are diagnosed each year, and new cancer cases are predicted to be 28 million per year in 2040.^1^ Among children with some forms of cancer, about one of a third is affected by a type of leukemia, most commonly acute lymphoblastic leukemia (ALL), which is characterized by an abnormal rapid proliferation of immature blood cells, blocking out the normal formation of blood cells in the bone marrow. Despite a relatively good prognosis approximately one fourth of the patients suffer from relapse and, consequently, a worse prognosis. Furthermore, drug resistance is a serious drawback of many existing anti-leukemia drugs.^2^ Continued research on the molecular aspects of leukemic cells has resulted in a greatly improved understanding, diagnosis, treatment and prevention of leukemia. It has been suggested that some cancer chemotherapeutics exert their effects by triggering apoptotic cell death. Accordingly, the induction of tumor cell apoptosis is used to predict tumor treatment response.^3^ Apoptosis is a selective process of physiological cell deletion that plays an essential role in the adjustment between cellular replication and death. Apoptotic signalling can proceed *via* two pathways, *i.e.*, *via* death receptors expressed on the plasma membranes of cells (extrinsic pathway), or, alternatively, *via* mitochondria, which contain several proteins that regulate apoptosis (intrinsic pathway).^4^ Still, the investigation of new potentially useful apoptosis-inducing therapeutic agents represents an important task for biomedical research.

**Fig. 1:**
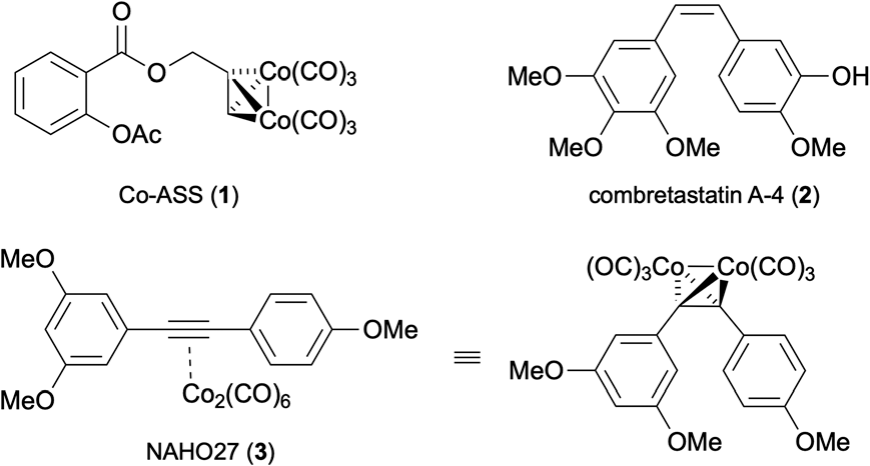
Structures of Co-ASS (1), combretastatin A-4 (2) and the designed complex NAHO27 (3) in two different representations.

While the undeniable success of *cis*-platin in the treatment of different cancer types has demonstrated the potential of metal-based anti-neoplastic agents, severe side effects and problems with drug resistance have prompted an intense search for other metal-based antitumor agents, and several metal-containing compounds based on titanium, germanium, rhodium, rhenium, gallium, gold, ruthenium, tin, cobalt or copper have shown promising activities and have even been included in clinical trials.^5^ In 1987, Hyama *et al.* first reported a new class of anti-neoplastic organometallic compounds based on a cobalt-complexed alkyne substructure.^6^ Later, Jung *et al.* showed that Co_2_(CO)_6_-complexed propargyl acetylsalicylate (Co-ASS, 1) (Fig. 1) exhibits high cytostatic activity on cells of skin and lung tumor origin.^7^ The complexation of the alkyne unit with a Co_2_(CO)_6_ fragment was proven to be essential for the cytostatic effect. Extensive studies by Gust and co-workers revealed that the cytostatic and apoptosis-inducing properties of 1 are associated with the inhibition of cyclooxygenase 1 and 2 (ref. 8) or, more fundamentally, with the regulation of NF-κB activity.^9^ In addition, there is indirect evidence that slow carbon monoxide (CO) release from the Co_2_(CO)_6_ unit might partly account for the observed biological effects of 1.^10^

In addition to Co-ASS (1) and analogues, several structurally distinct Co_2_(CO)_6_–alkyne complexes were found to possess remarkable levels of *in vitro* antitumor activity.^11^ As an example, Co_2_(CO)_6_-complexed diphenylacetylene exhibited superior antiproliferative activity (in comparison to Co-ASS) towards myeloid leukemia and acute lymphatic leukemia cell lines.^12^ Inspired by this evidence, we considered the trimethoxy-substituted complex 3 (Fig. 1) to be a promising candidate for the investigation of antitumor properties, as this organometallic compound would be structurally related to the natural antineoplastic agent combretastatin A-4 (2) which is a most promising small-molecule antitumor agent.^13^ Besides acting as a potent tubulin polymerisation inhibitor, this compound possesses high (and tissue-selective) antiangiogenic activity which might be a consequence of a yet unknown mechanism of biological action,^12^ and unlike other cell-cycle specific agents, it does not exhibit any hematopoietic side-effects *in vivo*. The *Z*-configuration of the stilbene unit in 2 is crucial for the observed antitumor effect, while isomerisation to the *trans*-isomer leads to a loss of activity and side-effects. The double bond in 2 plays a key constructive role (to assure the bent geometry) but is not involved in binding to the biological target (tubulin). Thus, the synthesis of *cis*-restricted analogues by modification of the olefin moiety is considered to be a promising approach for combretastatin A-4 structure optimization.^14^ Herein, we report the synthesis and detailed biological assessment of compound NAHO27 (3), a *cis*-locked structural analogue of 2, in which the Co_2_(CO)_6_–alkyne motif is used to keep the two aryl pharmacophores in the required 1,2-*cis* spatial orientation. We also describe the pronounced cytostatic effects of 3 in several cell cancer lines and provide an insight into the mechanism of biological action.

## Results

### Synthesis and chemical characterization of NAHO27 (3)

**Scheme 1:**
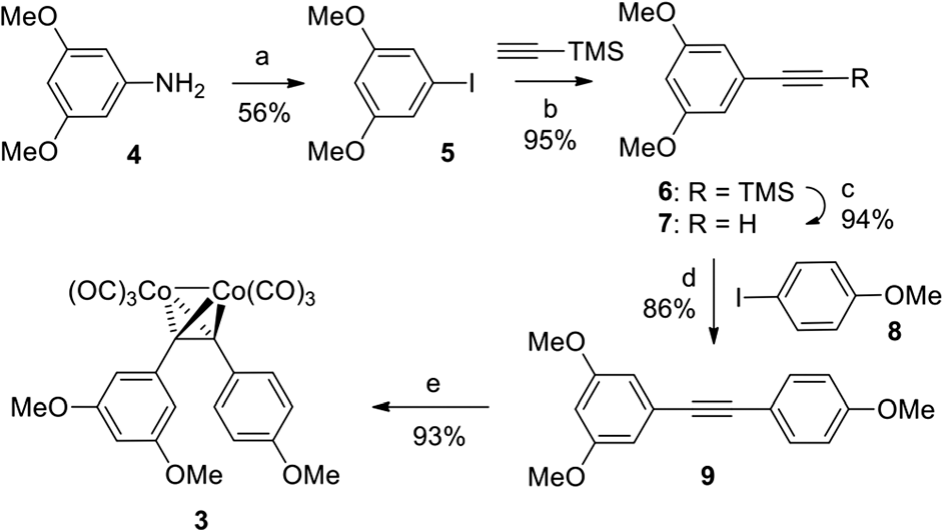
Reagents and conditions: a) *p*-toluenesulfonic acid monohydrate, NaNO_2_, MeCN/H_2_O, 10 °C, then aq. KI; b) Pd(PPh_3_)_2_Cl_2_ (5 mol%), CuI (4 mol%), PPh_3_ (2 mol%), NEt_3_, 50 °C; c) TBAF, THF, r.t.; d) same as (b) but at r.t.; e) Co_2_(CO)_8_, THF, r.t. TBAF = tetrabutylammonium fluoride; THF = tetrahydrofurane.

Complex 3 was synthesized in a straightforward five-step sequence starting from 3,5-dimethoxyaniline (4) and 4-iodoanisole (8) as is depicted in Scheme 1. Aniline 4 was first converted into the corresponding aryl iodide 5 using a Sandmeyer reaction.^15^ Subsequent Sonogashira coupling of 5 with trimethylsilyl acetylene followed by TBAF-mediated deprotection of the TMS-group provided the terminal alkyne 7.^16^ The latter was subjected to a second Sonogashira coupling with 4-iodoanisole 8 to form diarylalkyne 9,^17^ which upon treatment with dicobaltoctacarbonyl in THF provided the target complex 3 in 40% yield over 5 steps.

**Fig. 2:**
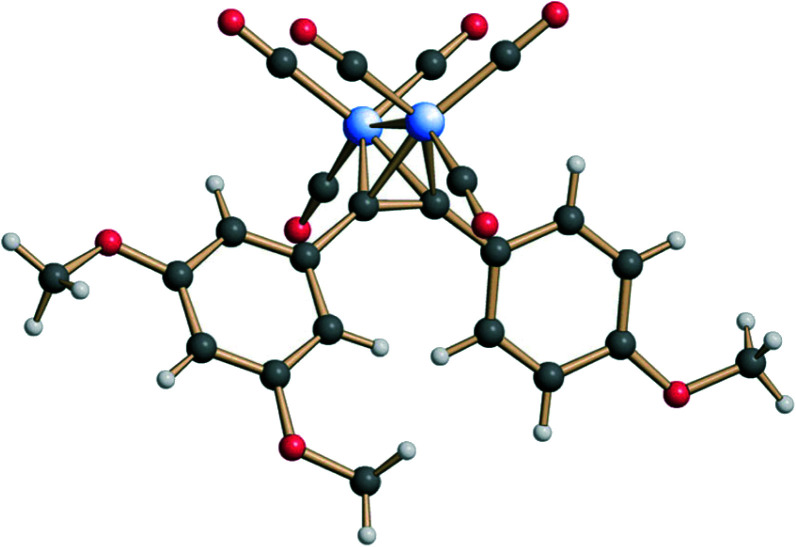
Structure of complex 3 in the crystalline state.

The stereostructure of complex 3 in the crystalline state was investigated by means of X-ray single crystal diffraction analysis (Fig. 2). The bend angle and the dihedral angle between the aryl fragments in 3 were determined as 108° and 36°, respectively (the analogous parameters for 2 are 83° and 60°).^18^ The distance between the oxygen atoms of the methoxy-groups from the neighboring aryl fragments in 3 equals 8.11 Å (6.70 Å for the analogous distance in 2). Thus, complex 3 differs from the natural stilbene 2 in its exact geometric parameters, but still displays the essential structural features, *i.e.*, the two non-coplanar *cis*-positioned methoxylated aryl fragments.

### Biological investigations

**Fig. 3:**
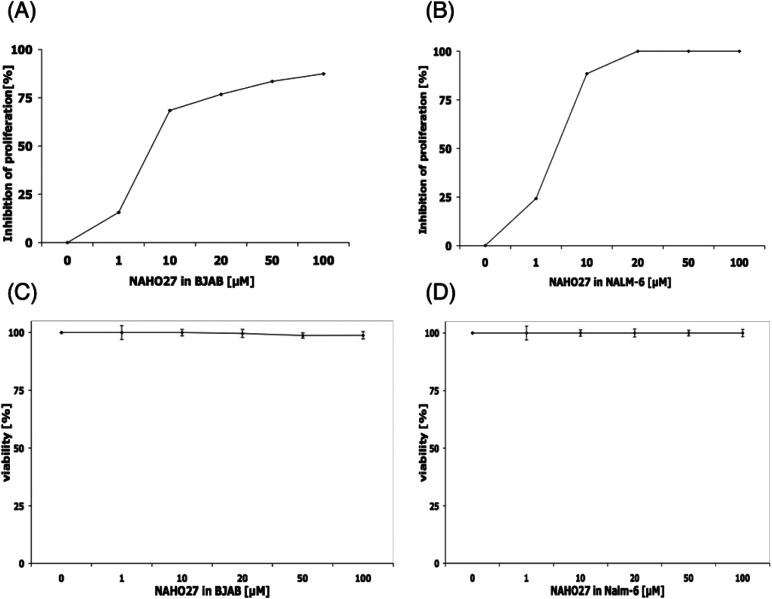
NAHO27 (3) inhibits proliferation of BJAB and Nalm-6 cells without causing undirected necrosis. Cells were either left untreated as control or incubated with different concentrations of 3. After 24 h, the cell proliferation of BJAB (A) and NALM-6 (B) cells was determined using the CASY®CellCounter + analyzer system. Inhibition of proliferation is given in % of control ± SD (*n* = 3). Direct membrane damage in BJAB (C) and NALM-6 cells (D) was determined by measurement of LDH release into the medium after 1 h of incubation with 3. Data are displayed as viability [%], and the values are given in % of control ± SD (*n* = 3).

We first examined the effect of compound 3 (NAHO27) on the cell growth of various cancer cell lines. Incubation of both BJAB and Nalm-6 cells with a concentration range of 1–100 μM of 3 led to a statistically significant decrease in cell viability after 24 h. The anti-proliferative effect of NAHO27 in Nalm-6 (Fig. 3B) cells was stronger than in BJAB cells (Fig. 3A). IC_50_ values were in the range of 1–10 μM (Nalm-6) and around 10 μM for BJAB, respectively. In order to exclude a possible necrotic effect of the agent, lactose dehydrogenase (LDA) was shown to be absent in culture supernatants of BJAB (Fig. 3C) and Nalm-6 (Fig. 3D) cells incubated 2 h with 1–100 μM of 3. LDA is an enzyme which is released by plasma membrane lysis and is characteristic for cell necrosis, while apoptotic cells maintain their membrane integrity.^19^

**Fig. 4:**
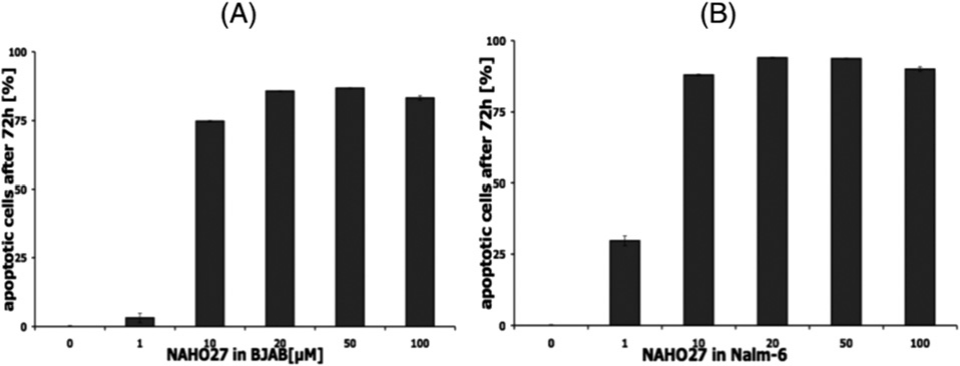
NAHO27 (3) induces apoptosis in leukemia and lymphoma cells. Cells were either left untreated as control or incubated with different concentrations of 3. After 72 h, DNA fragmentation in BJAB (A) and Nalm-6 (B) cells was determined by flow cytometric analysis. Values of DNA fragmentation are given in % of control ± SD (*n* = 3).

To prove that the cell growth inhibitory effect of NAHO27 (3) is associated with the induction of apoptosis, DNA fragmentation – a hallmark of apoptotic cells – was shown to increase in a dose-dependent manner in BJAB (Fig. 4A) as well as in Nalm-6 (Fig. 4B) cells. These data show that NAHO27 (3) significantly induces DNA fragmentation (up to 80%) in both BJAB and Nalm-6 cells with a half maximal concentration (AC_50_) between 7 and 10 μM.

**Fig. 5:**
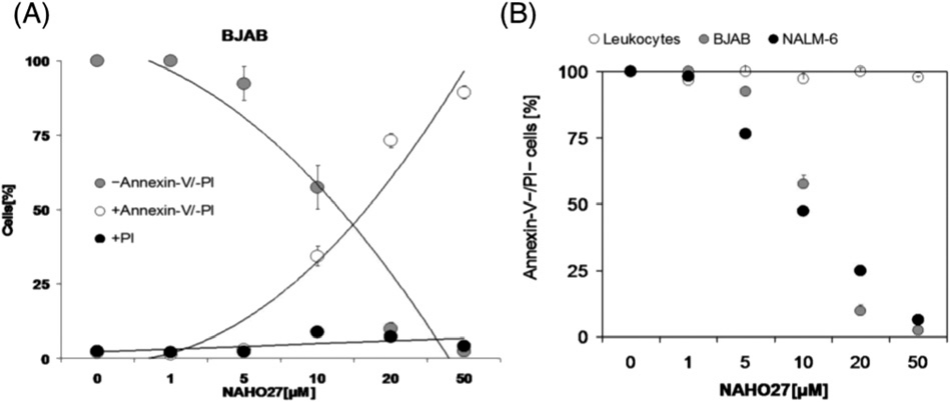
NAHO27 (3) acts selectively against malignant cells as reflected by induction of apoptotic cell death in BJAB and Nalm-6 cancer cells but not in healthy leukocytes. (A) Cell death in BJAB cells was classified as apoptotic (annexin-V+/PI−) and unspecific cell death (PI+) and compared with the frequency of vital cells. (B) BJAB, Nalm-6 cells and primary leukocytes obtained from a healthy person were either left untreated as controls or incubated with different concentrations of NAHO27. After 48 h, we determined the number of vital cells (annexin-V−/PI−). Values are given in % of control ± SD (*n* = 3).

Furthermore, we assessed the translocation of phosphatidylserine (PS) using annexin V and PI double staining. Early and late phase apoptotic BJAB cells were found to increase proportionally to the decrease of vital cells, while unspecific cell death was only minimal (Fig. 5A). The half-maximal lethal concentration, which reflects the ability to reduce the number of vital cells (annexin-V−/PI−) by 50% after 48 h of incubation with NAHO27, was determined to be around 10 μM for Nalm-6 cells and between 10 and 15 μM for BJAB cells, respectively (Fig. 5B). In addition, we investigated the selectivity of NAHO27 *ex vivo* and analyzed whether the compound was less active against normal, non-malignant cells. And indeed, the tested healthy leukocytes showed a constant rate of vital cells (Fig. 5B). Taken together, these data indicate that NAHO27 (3) specifically induces apoptosis in both lymphoma (BJAB cells) and leukemia cells (Nalm-6) while they do not affect normal leukocytes.

**Fig. 6:**
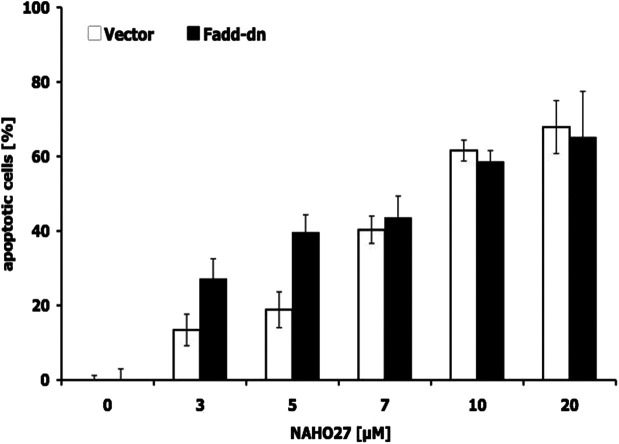
Apoptosis induction by NAHO27 (3) occurs independently from CD95/Fas. Vector- and FADD-dn-transfected BJAB cells were treated with different concentrations of the agent for 72 h. Then, DNA fragmentation was measured by flow cytometric analysis of cellular DNA content. Values are given as percentages of cells with hypodiploid DNA ± SD (*n* = 3).

To define the mechanism associated in NAHO27-induced apoptosis, first, the possible involvement of the CD95/Fas receptor was investigated using BJAB cells overexpressing a dominant-negative FADD (FADD-dn) mutant^22^ alongside control cells lacking the mutant. Treatment of both mutant and controls with different concentrations of NAHO27 indicated that the apoptosis induction is independent of CD95/Fas as shown by DNA fragmentation analysis (Fig. 6). Thus, the extrinsic pathway does not appear to play any significant role in the apoptosis induced by NAHO27.

**Fig. 7:**
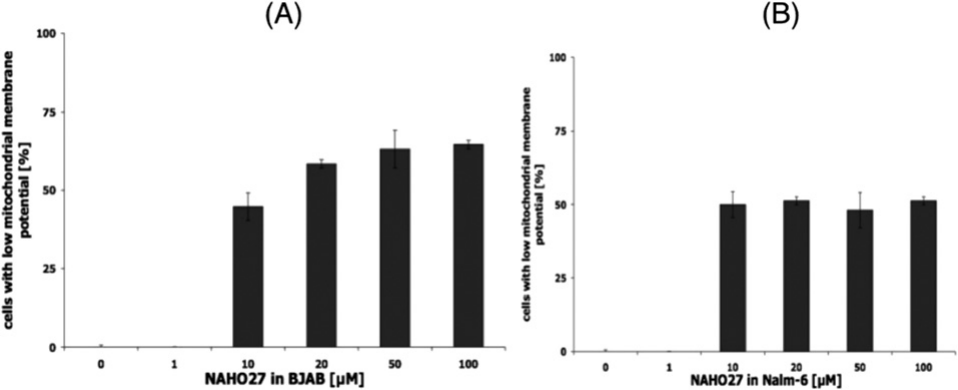
Effect of NAHO27 (3) on the mitochondrial membrane potential. BJAB (A) and Nalm-6 (B) cells were incubated with different concentrations of 3 for 48 h. Then, mitochondrial permeability transition was measured by flow cytometric analysis on the single cell level. Values of mitochondrial permeability transition are given as percentages of cells with low mitochondrial membrane potential ± SD (*n* = 3).

To probe whether NAHO27-induced apoptosis proceeds mainly *via* the mitochondrial (intrinsic) pathway, we used JC-1 (as a mitochondria-specific voltage-dependent dye)^20^ to investigate whether NAHO27 (3) is able to trigger mitochondrial membrane depolarization. Therefore, BJAB cells and Nalm-6 cells were treated with different concentrations of 3. JC-1 staining after 48 h of incubation followed by flow cytometric analysis of mitochondrial permeability transition revealed a concentration-dependent disruption of membrane potential of up to 70% for BJAB (Fig. 7A) and 55% for Nalm-6 (Fig. 7B) cells.

**Fig. 8:**
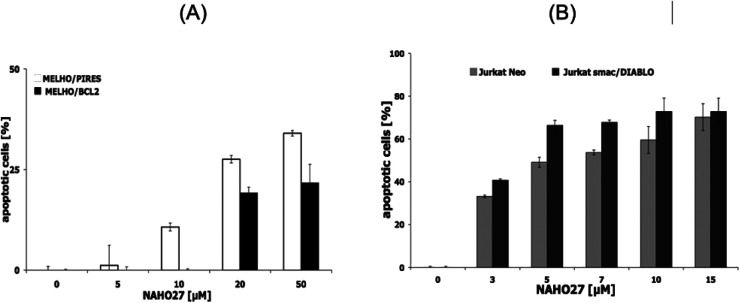
Involvement of Bcl-2 and smac/DIABLO in apoptosis induction by NAHO27 (3). (A) Multidrug-resistant Mel-HO-Bcl-2 melanoma cells (stable transfection and overexpression of Bcl-2) were treated with 3. For control, the vector-transfected Mel-HO clone (Mel-HO-pIRES) was used. DNA fragmentation was measured after 72 h by flow cytometric analysis. (B) Jurkat/smac cells (stable transfection and overexpression of smac/DIABLO) were treated with 3. For control, the vector-transfected Jurkat clone (Jurkat/neo) was used. DNA fragmentation was measured after 72 h by flow cytometric analysis of cellular DNA content ± SD (*n* = 3).

To additionally determine the role of Bcl-2 in NAHO27-induced apoptosis, Mel-HO-Bcl2 melanoma cell lines (transfected to overexpress Bcl-2) were incubated with different concentrations of NAHO27 (3). Likewise, to investigate the involvement of smac/DIABLO, Jurkat/smac, a leukemia cell line transfected to overexpress smac/DIABLO^21^ was also incubated with 3. Flow cytometric analysis revealed that the overexpression of Bcl-2 partially inhibits NAHO27-induced apoptosis (Fig. 8A), whereas smac/DIABLO overexpression promotes it (Fig. 8B).

**Fig. 9:**
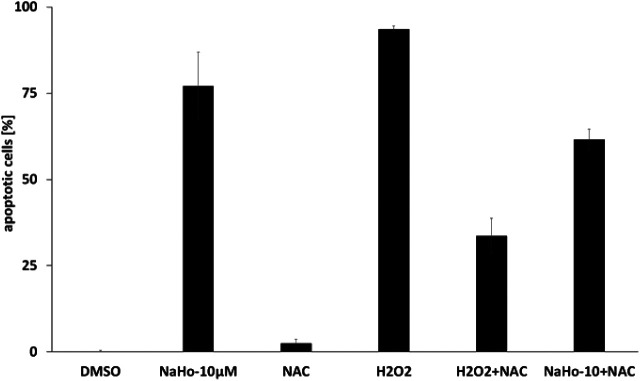
Dependence of apoptosis induced by NAHO27 (3) on the ROS pathway. Nalm-6 cells were treated with 3 in the presence of *N*-acetyl cysteine (NAC) as a ROS-suppressing agent. DNA fragmentation was measured after 72 h by flow cytometric analysis. For control, cells were also treated with NAC, H_2_O_2_ (as a ROS inducer) and a combination of both agents. DNA fragmentation was measured after 72 h by flow cytometric analysis of cellular DNA content ± SD (*n* = 3).

Using Nalm-6 leukemia cells we could also show that NAHO27-induced apoptosis partly proceeds *via* the ROS pathway as reflected by a significant suppression of apoptosis (from 77% to 62%) by the ROS inhibitor *N*-acetyl-cystein (NAC) (Fig. 9). While NAC itself does not induce apoptosis, the control shows its effectiveness in reducing H_2_O_2_-induced ROS.

**Fig. 10:**
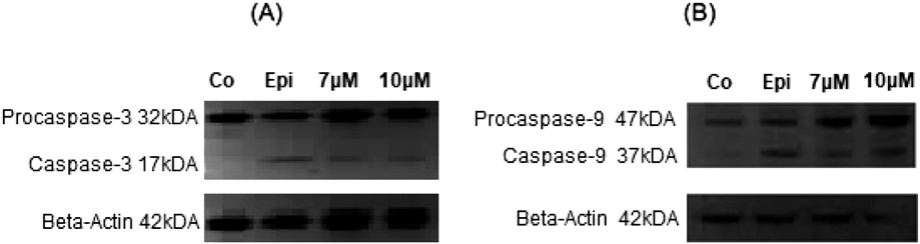
The activity of NAHO27 (3) is associated with a weak activation of caspases 3 and 9. BJAB cells were incubated with 7 and 10 μM of 3 and with 5 μM epirubicin as positive control. After 24 h of incubation, the cells were collected and lysed. 40 μg of cytosolic protein was separated by sodium SDS-PAGE and subjected to western blot analysis. Immunoblots developed with anticaspase-3 or -9 and anti-beta-actin are shown. The position of the 17 kDa active subunit of caspase-3 (A) and the 37 kDa (B) active subunit of caspase-9 are indicated. Equal loading and blotting was verified by detection of beta-actin (β-actin).

Furthermore, the activation of caspases was investigated by western blot analysis of NAHO27-treated BJAB cells. We found that procaspases 3 and 9 are (at least to a certain extend) processed to the active p17 subunit (C3) (Fig. 10A) and the active 37 kDa product (C9) (Fig. 10B).

**Fig. 11:**
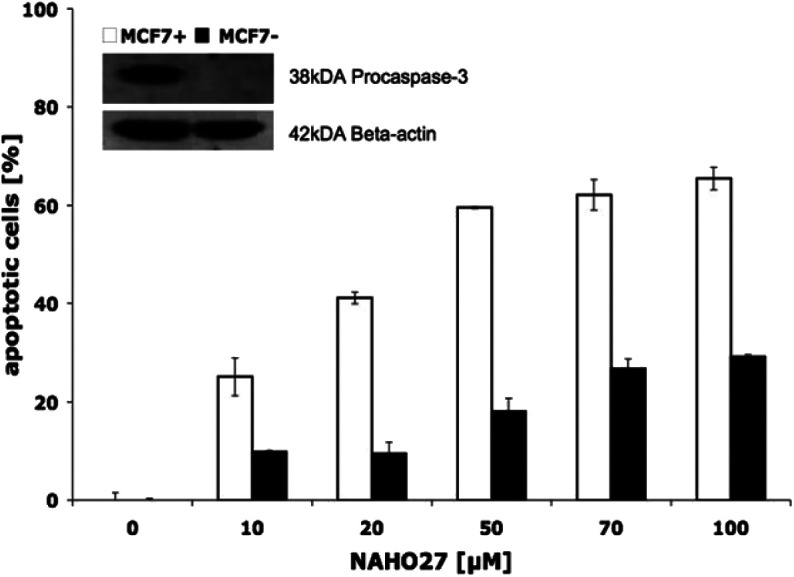
Apoptosis induction by NAHO27 (3) only partly depends on caspase-3. Caspase-3 deficient MCF7− cells were treated with 3. For control, the vector-transfected MCF7+ was used (stable transfection and overexpression of caspase-3). Caspase-3 negative and positive MCF7 cells were incubated with different concentrations of 3. After 96 h DNA fragmentation was determined by flow-cytometric analysis. Data are given in % hypoploidy (subG1), which reflects the number of apoptotic cells ± SD (*n* = 3). To demonstrate caspase-3 overexpression, 40 μg of cytosolic extracts of both cell lines were subjected to western blot analysis and incubated with an anti-caspase-3 antibody. Equal loading was verified using beta-actin.

Furthermore, by parallel treatment of caspase-3-deficient and normal MCF-7 breast cancer cells with different concentrations of NAHO27 we could show that the induced apoptosis depends only slightly on caspase-3 (Fig. 11).

**Fig. 12:**
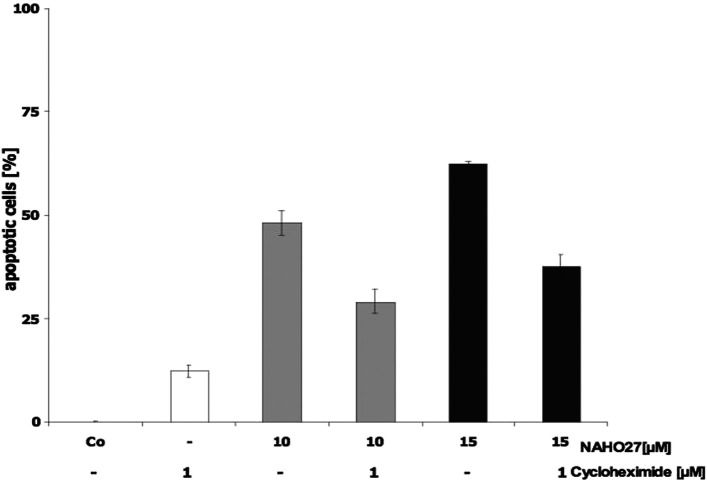
Cycloheximide impairs NAHO27-induced DNA fragmentation. BJAB cells were preincubated with 1 μM of cycloheximide. After 1 h, the cells were treated with NAHO27 (3) (10/15 μM). DNA fragmentation was determined after 72 h. Combination of cycloheximide with 3 results in a decrease in DNA fragmentation. Values are given as percentages of cells with hypodiploid DNA ± SD (*n* = 3).

To determine the possibility that new protein synthesis is required for NAHO27-induced apoptosis, BJAB cells were preincubated with cycloheximide (a protein synthesis inhibitor)^23^ for 1 h and then treated with NAHO27. Flow cytometric analysis after 72 h shows a partial inhibition of the DNA fragmentation (Fig. 12). This indicates that NAHO27-induced apoptosis also depends on the expression of new proteins.

**Fig. 13:**
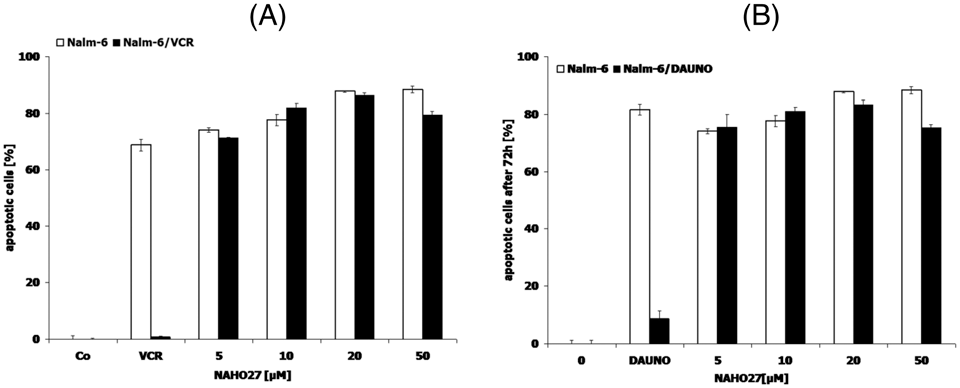
Activity of NAHO27 (3) against resistant leukemia cells. Drug-resistant cell lines ((A): Nalm-6/BeKa, (B): Nalm-6/LiKa) and the corresponding non-resistant cells Nalm-6 were incubated either with 3 or with the drugs to which the respective cells are tolerant. Vincristine was applied at a concentration of 20 nM and daunorubicin at 80 nM. Some cells of each cell line were left untreated as controls. DNA fragmentation was measured after 72 h by flow cytometric analysis. Values are given as percentages of cells with hypodiploid DNA ± SD (*n* = 3).

Using our previously developed cell lines resistant to selected chemotherapeutic agents,^24^ we investigated whether NAHO27 (3) is also effective against vincristine-, daunorubicin-, and doxorubicin-resistant cells. For this purpose, we incubated the vincristine- and daunorubicin-resistant cell lines Nalm-6/BeKa and Nalm-6/LiKa, respectively, with different concentrations of 3. Indeed, DNA fragmentation analysis showed that NAHO27 is also highly effective in inducing apoptosis in these resistant cell lines, which both show a co-resistance to anthracyclines (idarubicin, daunorubicin, doxorubicin, epirubicin), mitoxanthrone, fludarabine, vinca alkaloids (vincristine, vindesine, vinorelbine, vinblastine) and etoposide *in vitro*^25^ (Fig. 13A and B). Overexpression of the membrane drug transport protein p-glycoprotein, which is commonly observed in MDR cancer,^26^ has been detected in both Nalm/BeKa and Nalm/LiKa cells. To examine the mechanism by which NAHO27 is able to overcome resistance against these cell lines, we determined the expression of 84 apoptosis-relevant genes in BJAB cells after treatment with the agent and found that the harakiri gene is upregulated by 9%. Harakiri is a pro-apoptotic protein, which interacts with the death repressor protein Bcl-2.^27^

**Fig. 14:**
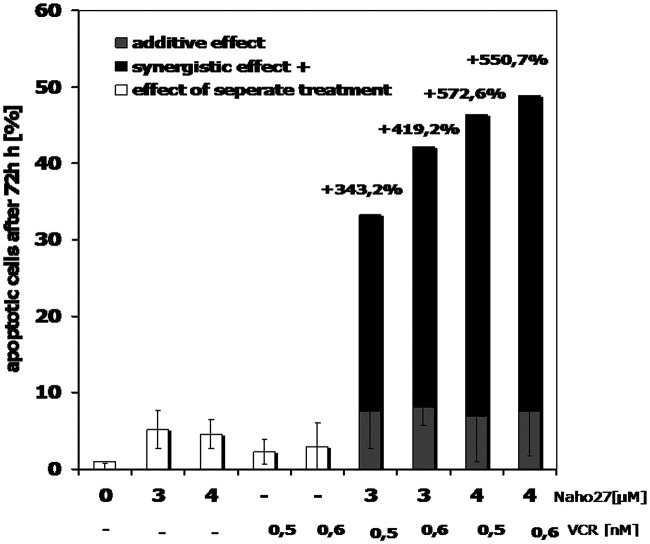
Synergistic activity of NAHO27 (3) and vincristine. BJAB cells were incubated either with 3 (3 and 4 μM), with vincristine (0.5 and 0.6 nM) or with combinations of both compounds. The agents were applied at very low concentrations to detect possible synergistic effects. As a control, some cells were left untreated. After 72 h, DNA fragmentation was measured by flow cytometric analysis. Values are given as percentages of cells with hypodiploid DNA ± SD (*n* = 3).

Finally, we investigated whether NAHO27 (3) exhibits synergistic effects with vincristine as a common anti-cancer drug. Therefore, BJAB cells were incubated with low concentrations of 3 in combination with low concentrations of vincristine. At these concentrations, both compounds were shown to cause only minimal cell death when applied individually. Noteworthy, a strong synergistic effect of up to 550% was observed (Fig. 14).

## Discussion

Apoptosis plays an important part in maintaining homeostasis and elimination of damaged cells,^28^ and many anti-cancer drugs act by causing the death of tumor cells through the induction of apoptosis.^29^ The purpose of the present study was to investigate the anti-tumor activity of the novel cobalt alkyne complex NAHO27 (3) on lymphoma (BJAB), leukemia (NALM-6, Jurkat), melanoma (Mel-HO) and breast cancer cells (MCF7) *in vitro*. Noteworthy, this compound (3) was designed as a combretastatin A4-related molecule and shown to be synthetically easily accessible.

The results presented above demonstrate a strong and dose-dependent inhibition of proliferation in different cancer cell lines upon treatment with NAHO27 (3) associated with high levels of DNA fragmentation. Although detection of DNA fragmentation in cancer cells is a sensitive indication of the apoptotic process, it is not a specific signature exclusive to this type of cell death as necrotic cells can also exhibit DNA fragmentation.^30^ However, using the translocation of phosphatidylserine as an additional experimental method, we were able to rule out a possible necrotic effect of NAHO27.

The goal of cancer chemotherapy is to destroy all cancer cells while causing as little damage as possible to normal tissues.^31^ However, a downside of many clinically used cytostatic drugs are the severe side effects on healthy cells. Especially leukocytes are often damaged. With this in mind, we tested the selectivity of our agent on normal cells and could not observe any significant toxicity against healthy leukocytes *ex vivo*. This suggests a high selectively of NAHO27 (3) against malignant cells. However, because the sensitivity and tolerance of cells *in vivo* and *ex vivo* are different, this result must be viewed with due caution.

The promising *in vitro* activity of NAHO27 (3) prompted us to investigate its mechanism of apoptosis induction. In this context we could show that NAHO27-induced apoptosis occurs independently from the death receptor CD95/Fas. This result points towards the intrinsic or mitochondrial pathway of apoptosis, which is associated with changes in the permeability of the outer mitochondrial membrane and the collapse of the membrane potential.^35^ And indeed, we could show an early disruption of the mitochondrial membrane potential in cancer cells treated with NAHO27. While the activation of caspase-3 correlates with the activation of caspase-9, which acts as a key initiator of this pathway, the mitochondrial outer membrane permeabilization is governed by anti- and pro-apoptotic members of the Bcl-2 protein family.^36^ The observed dependence of NAHO27-induced apoptosis on overexpression of Bcl-2 (an anti-apoptotic protein of the intrinsic pathway) and on overexpression of smac/DIABLO (a pro-apoptotic mitochondrial protein)^37^ further confirmed that NAHO27-induced apoptosis follows the intrinsic pathway – in line with the observation that apoptosis induction is slightly suppressed in the presence of NAC as a ROS-suppressing agent. Using cycloheximide (an inhibitor of transcriptional-steered apoptosis)^38–40^ we could show that NAHO27-induced apoptosis is influenced by transcriptional or translational changes. And by means of gene expression analysis, we detected a 9-fold up-regulation of the pro-apoptotic harakiri gene in lymphoma cells treated with NAHO27. Whether part of the activity of NAHO27 relates to its ability to release carbon monoxide (up to 1 equivalent after 100 h; see the ESI†) must be investigated in future studies.

Another serious problem in cancer therapy is the frequent occurrence of resistance against the drugs used, and several MDR proteins responsible for this resistance have been identified.^31,32^ For this reason, we also tested NAHO27 against our resistant leukemia cell lines Nalm/BeKa and Nalm/LiKa, which are known to overexpress p-glycoprotein (MDR1 transporter), a membrane-associated protein responsible for the active excretion of drugs from cancer cells to prevent them from binding their targets.^33,34^ The fact that NAHO27 proved to be effective in inducing apoptosis also in these cells suggests an ability of this agent to overcome multiple drug resistance in cancer cells. Finally, we evaluated the use of NAHO27 (3) in a so-called combination therapy, which is a promising strategy to combat drug resistance in cancer treatment. The idea is to combine drugs with different mechanisms and sites of action to achieve a complementary (synergistic) rather than merely additive effect and to minimize side effects.^31^ We could indeed demonstrate that NAHO27 shows a strong synergistic effect (550%) with co-administered vincristine at low concentration.

## Conclusion

In conclusion, we have demonstrated that the organometallic combretastatin A-4 analog NAHO27 (3), readily available in only few synthetic steps, induces a strong apoptotic response in leukemia and other malignant tumor cells, which is mainly proceeding *via* the intrinsic (mitochondrial) pathway. Moreover, this agent was shown to overcome multidrug resistance in leukemia cells *in vitro*, suggesting that its mechanism of action differs from these drugs. In any case, due to its high activity, which also shows a strong synergy in combination with vincristine, NAHO27 (3) must be considered as an interesting molecule with a potential for cancer chemotherapy, which may deserve further pre-clinical evaluation.

## Author contributions

A. P. and H.-G. S. initiated research; N. H. and L. H. performed chemical syntheses and CO-release studies; L. A. O., L. K., B. K. and C. F. performed biochemical investigations; J.-M. N. performed X-ray crystal structure analyses; L. H., N. S., C. F., A. P. and H.-G. S. wrote the manuscript.

## Conflicts of interest

There are no conflicts to declare.

## Supplementary Material

MD-013-D2MD00144F-s001

MD-013-D2MD00144F-s002
