# Supplementary material for: An organometallic analogue of combretastatin A-4 and its apoptosis-inducing effects on lymphoma, leukemia and other tumor cells in vitro
Source: RSC Med Chem. 2022 Jun 30;13(9):1044–51. doi: 10.1039/d2md00144f (PMC9491352; doi:10.1039/d2md00144f)
Supplement: MD-013-D2MD00144F-s001 [file MD-013-D2MD00144F-s001.pdf]

Electronic Supplementary Material (ESI) for RSC Medicinal Chemistry.
This journal is © The Royal Society of Chemistry 2022

Supporting Information

An organometallic analogue of combretastatin A-4 and its apoptosis-inducing effects on lymphoma, leukemia and other tumor cells in vitro

Liliane Abodo Onambele, Natalie Hoffmann, Lisa Kater, Lars Hemmersbach, Jörg-Martin Neudörfl, Nicolai Sitnikov, Benjamin Kater, Corazon Frias, Hans-Günther Schmalz,* and Aram Prokop*

Experimental Part 1 (Chemistry)

General experimental conditions

All moisture sensitive reactions were carried out under argon atmosphere using Schlenk technique. Glassware was flame-dried under vacuum (<1 mbar) and allowed to cool under an argon atmosphere. Syringes, needles and transfer cannulas were dried in an oven at 100 °C and flushed with argon directly prior to use. Solvents were purified and dried according to standard procedures. Flash chromatography was performed using silica 60 (0.04 – 0.063 mm, 230-400 mesh, ASTM) supplied by *Merk. NMR spectroscopy:* ^{1}H and ^{13}C NMR spectra were recorded on a *Bruker* DPX 300 or an Avance AVIII-HD 500 instrument at room temperature. Chemical shifts (δ) are given in ppm relative to the solvent reference as an internal standard (^{1}H NMR: δ 7.26 ppm for $CDCl_3$, δ 7.15 ppm for C_6H_6 and δ 3.31 ppm for CD_3OD; ^{13}C NMR: δ 128.02 ppm for C_6H_6, δ 77.16 ppm for $CDCl_3$; δ 49.00 ppm for CD_3OD). The assignments of ^{1}H NMR are supported by H,H-COSY, HMQC(HSQC), and HMBC spectra. Carbon multiplicity assignment is based on APT or DEPT spectra. *Fourier transform infrared spectroscopy (FT-IR):* IR spectra were recorded on a *Perkin Elmer* FT-IR Paragon 1000 spectrometer using Fourier transform infrared (FTIR) multiple-point attenuated total reflection (ATR) technique. Absorption bands are given in wave numbers ($\tilde{\nu}$, cm^{-1}). Intensive bands are marked with (s), medium with (m), weak with (w). Broad bands are marked as (br). In all cases a solution of the sample compound in chloroform was used for film preparation. *Melting points* (*mp*) were measured on a *Büchi* B-545 melting point apparatus. *Mass spectrometry:* GC-MS experiments were carried out on an *Agilent* HP6890 System with mass detector (MSD) 5937 N. Low-resolution mass spectra (EI, 70 eV) were recorded on a *Finnigan* Incos 50 Galaxy System (DIP-MS). High-resolution mass spectra (EI, 70 eV) were recorded on a *Thermo Scientific Exactive GC* instrument. *Single crystal X-ray diffraction* studies were performed on a Nonius Kappa CDD diffractometer. *Elemental analyses* were performed on an *Elementar Vario EL* apparatus.

3,5-Dimethoxy-1-iodobenzene (5)

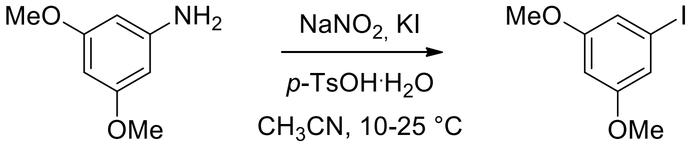

4 **5**

According to a modified procedure,[1] 3,5-dimethoxyaniline **4** (1.84 g, 12 mmol, 1.0 eq) was added at r.t. under vigorous stirring to a solution of *para*-toluenesulfonic acid monohydrate (6.85 g, 36 mmol, 3.0 eq) in acetonitrile (50 mL). The resulting suspension was cooled to 10 °C and solutions of sodium nitrite (1.66 g, 24 mmol, 2.0 eq) in water (5 mL) and potassium iodide (4.98 g, 30 mmol, 2.5 eq) in water (8 mL) were sequentially added. The reaction mixture was stirred 15 min at 10 °C and then at r.t. until full conversion was detected by TLC. The solution was diluted with water (100-150 mL) and brought to pH 9 with sat. aq. $NaHCO_3$ (30 mL). The excess of iodine was removed by addition of sat. aq. $Na_2S_2O_3$ (30 mL). The resulting mixture was extracted with methyl *tert*-butyl ether (3×60 mL) and the combined organic extracts were dried over $MgSO_4$. The solvent was evaporated under reduced pressure to give a brown oil which was subjected to column chromatography on silica gel (CyHex/EtOAc 7/1) to yield the iodide **5** (1.78 g, 6.75 mmol, 56%) as a white solid. **R_f** (SiO_2, CyHex/EtOAc 7/1): 0.28. **M.p.** 72.1 °C. **^{1}H NMR** (300 MHz, $CDCl_3$): δ = 3.74 (s, 6H, OCH_3), 6.38 (t, 1H, H-4), 6.38 (d, 2H, H-2, H-6). **^{13}C NMR** (75 MHz, $CDCl_3$) δ = 55.5 (OCH_3), 94.1 (C-1), 100.7 (C-4), 115.8 (C-2, C-6), 161.1 (C-3, C-5). **IR** (ATR): $\tilde{\nu}$ [cm^{-1}] = 2961 (w), 2926 (w), 2366 (w), 2326 (w), 1736 (w), 1650 (w), 1575 (s, br.), 1470 (m), 1453 (m), 1424 (m), 1295 (m), 1250 (w), 1199 (m), 1162 (m), 1050 (w), 1032 (s), 983 (w), 940 (w), 852 (m), 819 (w), 773 (w), 682 (w). **GC-MS** m/z (%) = 264 (72, [M]$^+$), 221 (3), 138 (100), 122 (8), 109 (29), 95 (19), 78 (25), 65 (15), 52 (8), 41 (8). **Elemental analysis** (%, calculated/found): C (36.39/36.41), H (3.44/3.46). The spectroscopic data are in accordance with the literature.[1]

((3,5-Dimethoxyphenyl)ethynyl)trimethylsilane (6)

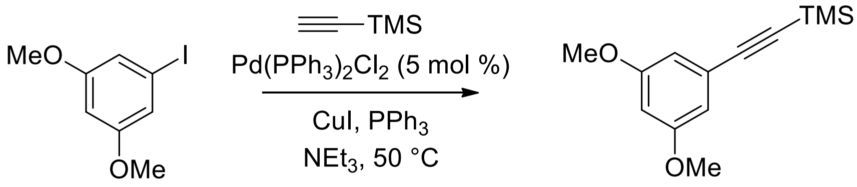

5 **6**

According to a modified procedure,[2] to a solution of aryl iodide **5** (1.7 g, 6.44 mmol, 1.0 eq) in triethylamine were added under argon atmosphere $Pd(PPh_3)_2Cl_2$ (226 mg, 0.322 mmol, 0.05 eq), copper iodide (49 mg, 0.258 mmol, 0.04 eq) and triphenylphosphine (34 mg, 0.129 mmol, 0.02 eq), and the resulting suspension was stirred at r.t. for 15 min. Trimethylsilylacetylene (1.0 mL, 7.08 mmol, 1.1 eq) was added dropwise and the reaction mixture was allowed to stir at r.t. for 48 h (full conversion detected by TLC). After filtration through a short pad of silica gel the solvent was removed under reduced pressure and the resulting green-brownish solid was subjected to column chromatography on silica gel (CyHex/EtOAc 20/1) to give **6** (1.43 g, 6.1 mmol, 95%) as a light-brown solid. **R_f** (CyHex/EtOAc 20/1): 0.28. **M.p.** 60.0 °C. **^{1}H NMR** (300 MHz, $CDCl_3$): δ = 0.23 (s, 9H, $Si(CH_3)_3$), 3.76 (s, 6H, OCH_3), 6.42 (t, 1H, H-4), 6.60 (d, 2H, H-2, H-6). **^{13}C NMR** (75 MHz, $CDCl_3$) δ = - 0.04 ($Si(CH_3)_3$), 55.4 (OCH_3), 93.7 (C≡*C*TMS), 102.2 (C-4), 105.0 (*C*≡CTMS), 109.6 (C-2, C-6), 124.3 (C-1) 161.1 (C-3, C-5). **IR** (ATR): $\tilde{\nu}$ [cm^{-1}] = 2960 (w, br), 2326 (w), 2159 (w), 1740 (w), 1694 (w), 1596 (m, br.), 1560 (w), 1540 (w), 1503 (w), 1453 (w, br.), 1420 (w), 1346 (w), 1326 (w), 1293 (w), 1248 (m), 1205 (m), 1157 (m), 1064 (m), 976 (w), 844 (m, br.), 760 (w), 680 (w). **GC-MS** m/z (%) = 234 (34, $[M]^+$), 219 (100), 205 (1), 189 (2), 176 (2), 161 (10), 131 (3), 109 (5), 73 (4), 43 (2). **Elemental analysis** (%, calculated/found): C (66.62/66.55), H (7.74/7.67).
The spectroscopic data are in accordance with the literature.[2]

3,5-Dimethoxy-1-ethynylbenzene (7)

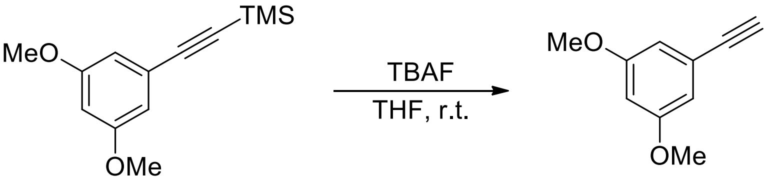

6 **7**

According to a modified procedure,[2] to a solution of compound **6** (1.2 g, 5.12 mmol, 1.0 eq) in anhydrous THF (25 mL) was added dropwise under argon atmosphere a 1 M solution of tetrabutylammonium fluoride in THF (1.8 mL, 1.80 mmol, 0.35 eq). The mixture was stirred at r.t. for 10 min followed by filtration through a short pad of silica gel. The solvent was removed under reduced pressure and the residue was subjected to column chromatography on silica gel (CyHex/EtOAc 3/1) to yield **7** (782 mg, 4.82 mmol, 94%) as a white solid. **R_f** (CyHex/EtOAc 3/1): 0.36. **^{1}H NMR** (300 MHz, $CDCl_3$): δ = 3.02 (s, C≡CH), 3.76 (s, 6H, OCH_3), 6.45 (t, 1H, H-4), 6.63 (d, 2H, H-2, H-6). **IR** (ATR): $\tilde{\nu}$ [cm^{-1}] = 3280 (w), 3269 (w), 2833 (w), 1590 (m), 1455 (m), 1419 (m), 1343 (w), 1321 (m), 1295 (w), 1250 (w), 1204 (m), 1157 (s), 1064 (m), 941 (w), 864 (m), 829 (m), 670 (w). **GC-MS** m/z (%) = 162 (100, $[M]^+$), 152 (5), 133 (20), 119 (14), 102 (23), 89 (15), 76 (12), 65 (10), 50 (9), 39 (3). The spectroscopic data are in accordance with the literature.[2]

1,3-Dimethoxy-5-((4'-methoxyphenyl)ethynyl)benzene (9)

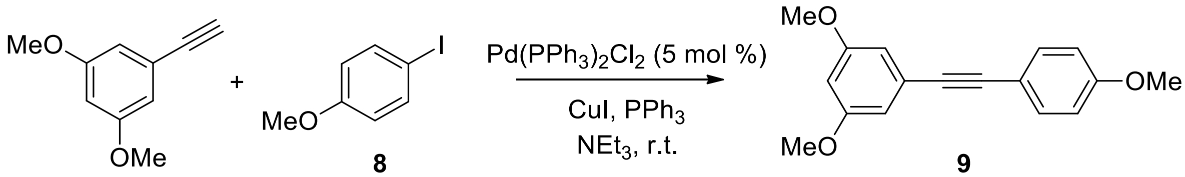

7 **8** **9**

According to a modified procedure,[3] to a solution of iodoanisole (**8**) (679 mg, 2.9 mmol, 1 eq) in triethylamine (35 mL) were added under argon atmosphere $Pd(PPh_3)_2Cl_2$ (102 mg, 0.145 mmol, 0.05 eq), copper iodide (22 mg, 0.116 mmol, 0.04 eq) and triphenylphosphine (15 mg, 0.058 mmol, 0.02 eq) and the resulting suspension was stirred at r.t. for 15

min. Then, a solution of alkyne **7** (470 mg, 2.9 mmol, 1 eq) in triethylamine (20 mL) was added dropwise and the mixture was allowed to stir at r.t. for 4 h (full conversion) followed by filtration through a short pad of silica gel. The solvent was removed under reduced pressure and the residue was subjected to column chromatography on silica gel (CyHex/EtOAc 5/1) to give **9** (668 mg, 2.49 mmol, 86%) as a yellow oil. **R$_f$** (CyHex/EtOAc 5/1): 0.4. **^{1}H NMR** (300 MHz, $CDCl_3$): δ = 3.78 (s, 6H, OCH_3), 3.81 (s, 3H, OCH_3), 6.23 (t, 1H, H-2), 6.65 (d, 2H, H-4, H-6), 6.85 (d, 2H, H-3', H-5'), 7.45 (d, 2H, H-2', H-6'). **^{13}C NMR** (75 MHz, $CDCl_3$) δ = 53.0 (OCH_3 at C-4'), 55.4 (OCH_3 at C-1, C-3), 88.1 and 89.0 (C≡C), 101.5 (C-2), 109.2 (C-4, C-6), 114.0 (C-3', C-5'), 115.2 (C-1'). 124.9 (C-5), 133.1 (C-2', C-6'), 159.7 (C-4'), 160.5 (C-1, C-3). **IR** (ATR): $\tilde{\nu}$ [cm^{-1}] = 3000 (w), 2957 (w), 2836 (w), 2206 (w), 2046 (w), 1587 (s, br), 1507 (s), 1455 (m), 1418 (m), 1356 (m), 1288 (m), 1247 (s), 1204 (s), 1170 (m), 1154 (s), 1120 (w), 1029 (m), 990 (w), 926 (w), 830 (m), 806 (w), 680 (w). **GC-MS** m/z (%) = 268 (100, $[M]^+$), 253 (22), 239 (7), 225 (8), 210 (4), 195 (6), 181 (4), 167 (5), 152 (10), 139 (7), 121 (12), 108 (5), 91 (6), 78 (8), 65 (7), 51 (3), 39 (5). The spectroscopic data are in accordance with the literature.[3]

Hexacarbonyl[1,3-dimethoxy-5-((4'-methoxyphenyl)ethynyl)-benzene]dicobalt (3)

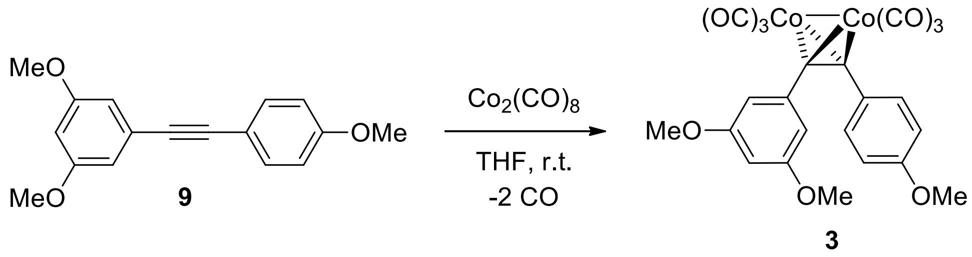

To a stirred solution of alkyne **9** (220 mg, 0.82 mmol, 1 eq) in anhydrous THF (8.2 mL) was added under argon atmosphere dicobaltoctacarbonyl (421 mg, 1.23 mmol, 1.5 eq) (caution: carbon monoxide evolution!) and the mixture was stirred at r.t. for 1 h. Silica gel (1 g) was added and the solvent was removed under reduced pressure. The residue was subjected to column chromatography on silica gel (CyHex/EtOH 10/1) to yield complex **3** (423 mg, 0.763 mmol, 93%) as dark red-brown crystals. **R$_f$** (SiO_2, CyHex/EtOAc 10/1): 0.38. **M.p. =** 69.7 °C. **^{1}H NMR** (500 MHz, acetone-d_6): δ = 3.82 (s, 6H, 2 x OCH_3), 3.86 (s, 3H, OCH_3), 6.56 (s, 1H, H-2), 6.80 (s, br., 2H, H-4, H-6), 7.04 (s, br., 2H, H-2', H-6'), 7.65 (s, br., 2H, H-3', H-5'). **^{13}C NMR** (125 MHz, acetone-d_6) δ = 55.7 (OCH_3 at C-1, C-3), 55.8 (OCH_3 at C-1'), 100.6 (C-2), 108.1 (C-4, C-6), 115.5 (C-3', C-5'), 130.3 (C-5), 131.5 (C-2', C-6'), 141.3 (C-4'), 160.9 (C-1'), 162.2 (C-1, C-3). **IR** (ATR): $\tilde{\nu}$ [cm^{-1}] = 2087 (w, CO), 2045 (m, CO), 2012 (m, CO), 1994 (w, CO). **MS** HR-EI Calc.: $[M-CO]^+$ = 525.9504 amu, found.: 525.9501 amu. (LR-EI, 70 eV) m/z (%) = 554 (2, $[M]^+$), 526 (4, $[M]^+$ - CO), 498 (4, $[M]^+$ - 2CO), 470 (14, $[M]^+$- 3CO), 422 (21, $[M]^+$ - 4CO), 414 (57, $[M]^+$ - 5CO), 386 (36, $[M]^+$ - 6CO), 357 (9), 327 (18), 268 (100, $[M]^+$ - $Co_2(CO)_6$), 258 (82), 255 (19), 236 (10), 225 (23), 193 (22), 169 (36), 152 (14), 134 (26), 118 (25). **Elemental analysis** (%, calculated/found): C (49.84/49.71), H (2.91/2.98).

NMR spectra of Hexacarbonyl[1,3-dimethoxy-5-((4'-methoxyphenyl)-ethynyl)-benzene]dicobalt (3)

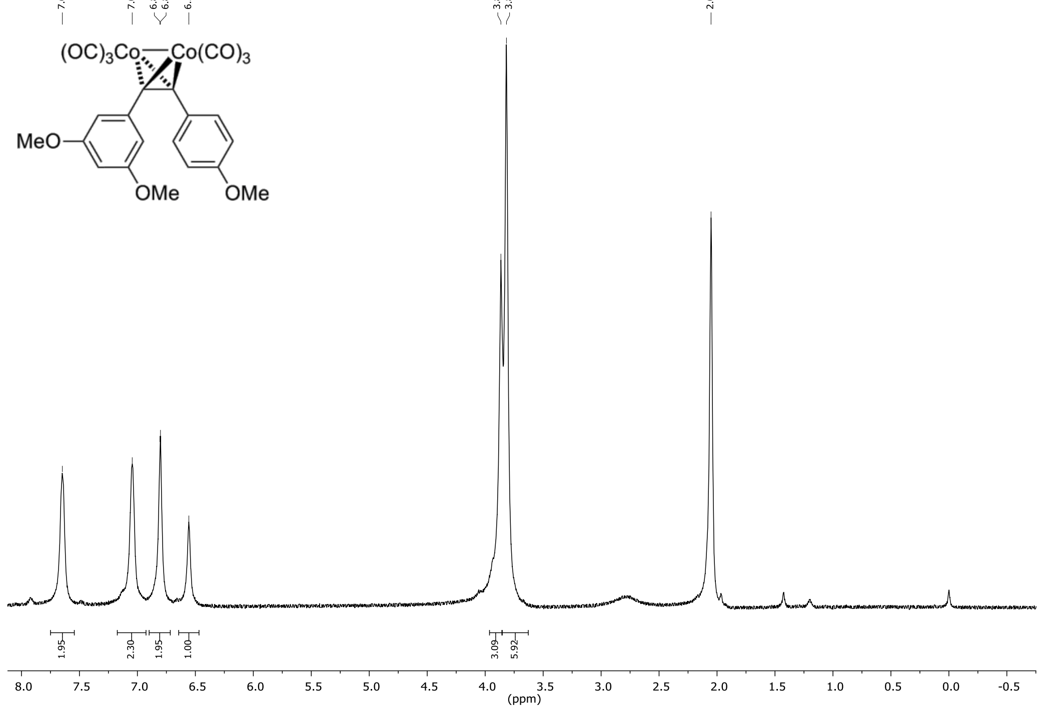

^{1}H NMR (500 MHz, acetone-d_6)

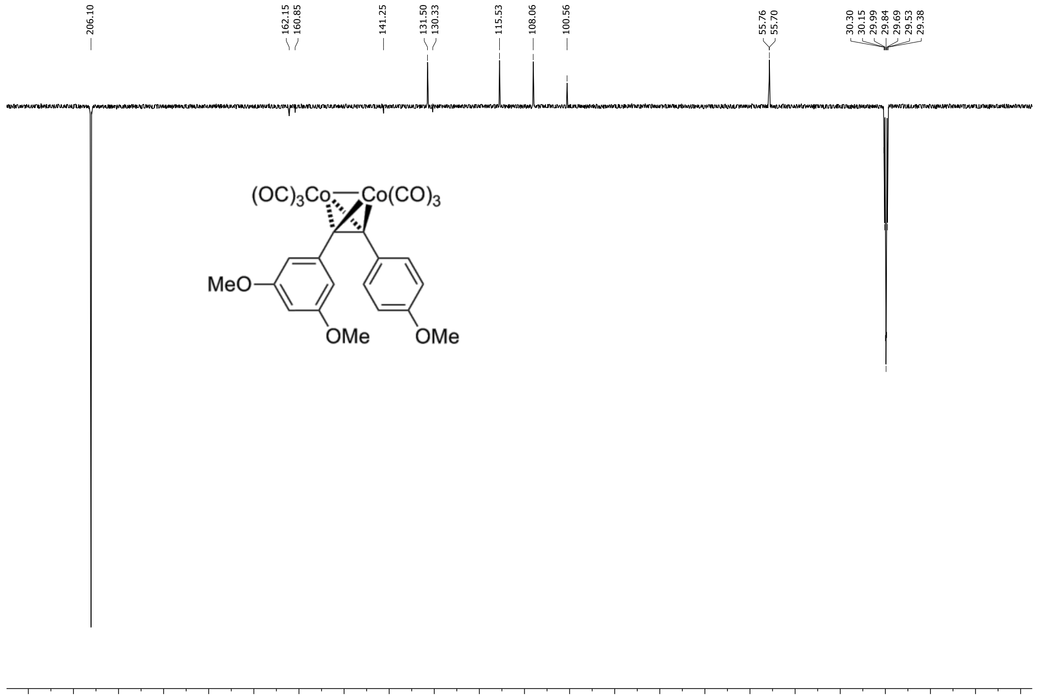

^{13}C NMR (125 MHz, acetone-d_6)

Crystal data and structure refinement for NaHo27 (3)

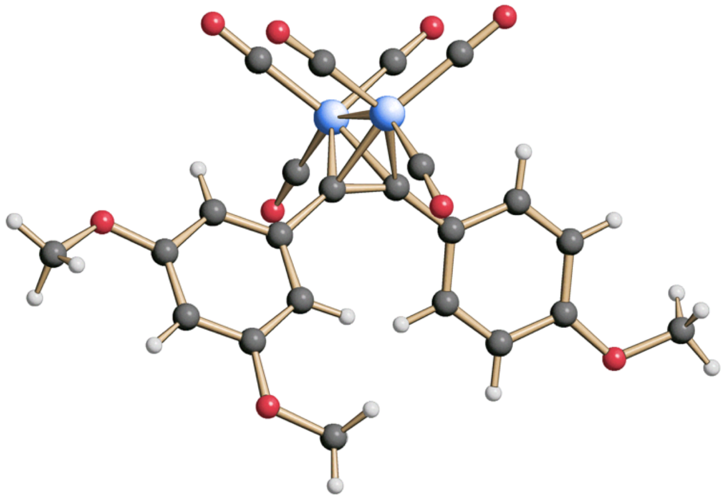

Empirical formula	$C_{23}H_{16}Co_2O_9$
Formula weight	554.22
Temperature	100(2) K
Wavelength	0.71073 Å
Crystal system, space group	triclinic, P1
Unit cell dimensions	a = 8.356(3) Å alpha = 85.039(15)°.
	b = 11.050(5) Å beta = 72.36(2)°.
	c = 13.470(5) Å gamma = 86.48(3)°.
Volume	1180.1(7) Å^3
Z, Calculated density	2, 1.560 Mg/m^3
Absorption coefficient	1.455 mm^{-1}
F(000)	560
Crystal size	.3 x .2 x .1 mm^3
Theta range for data collection	2.53 to 27.00 deg.
Limiting indices	-7<=h<=9, -5<=k<=14, -16<=l<=12
Reflections collected / unique	2916 / 2566 [R(int) = 0.0321]
Reflection observed	[I>2s(I)] 1220
Completeness to theta = 27.00	49.8%
Absorption correction	None
Refinement method	Full-matrix least-squares on F^2
Data / restraints / parameters	2566 / 0 / 310
Goodness-of-fit on F^2	0.804
Final R indices [I>2sigma(I)]	R1 = 0.0416, wR2 = 0.0797
R indices (all data)	R1 = 0.1134, wR2 = 0.0952
Largest diff. peak and hole	0.241 and -0.206 e.A^{-3}

CCDC 2144367 (**3**) contains the supplementary crystallographic data. The data can be obtained free of charge from The Cambridge Crystallographic Data Centre via www.ccdc.cam.ac.uk/structures

In situ quantification of CO release from NAHO27 (3)

A *Thermo Scientific Trace 1300* headspace gas chromatograph equipped with a *TriPlus RSH* autosampler was used. Detector type: thermal conductivity detector (TCD). Column: Shin carbon ST 100/120 1.0 mm × 2 m 1/16" OD silico. Software: *Chromeleon®* 7 Data System. Gas-carrier: helium; flow: 15 mL/min; injector temperature: 200 °C; split flow: 150 mL/min; split rate: 10; detector temperature: 200 °C. Method description: 0 – 2.5 min 35 °C, then to 70 °C with 20 °C/min rate, then 1 min at 70 °C, then to 35 °C with 20 °C/min rate, then 1 min at 35 °C. Injection volume: 50 µL.

Calibration: Eight headspace vials (BGB Analytics, cat. No. 200410-F, 10 mL) were filled with DMSO (0.2 mL) and phosphate buffer (0.1 m, pH = 7.4, 1.0 mL). Subsequently, the vials were closed with gas-tight silicon/PTFE septa crimp caps (BGB Analytics, cat. No. 20030500). Then, a defined gas volume was substituted by CO (0.00 mL, 0.05 mL, 0.10 mL, 0.25 mL, 0.50 mL, 1.00 mL, 1.50 mL, 2.00 mL). After equilibrating the vials for 10 min at 37 °C the composition of the gas phase was determined by headspace GC. These measurements were repeated three times and a calibration curve was generated.

In situ quantification of the CO release from NAHO27: The complex (36 µmol) was dissolved in DMSO (0.2 mL) and phosphate buffer (0.1 m, pH = 7.4, 1.0 mL). The mixture was stirred at 37 °C and the amount of released CO assessed through headspace GC using the previously recorded calibration curve.
The resulting graph (see Figure below) shows the liberation of ca. 1 equivalent of CO from NAHO27 after 100 h.

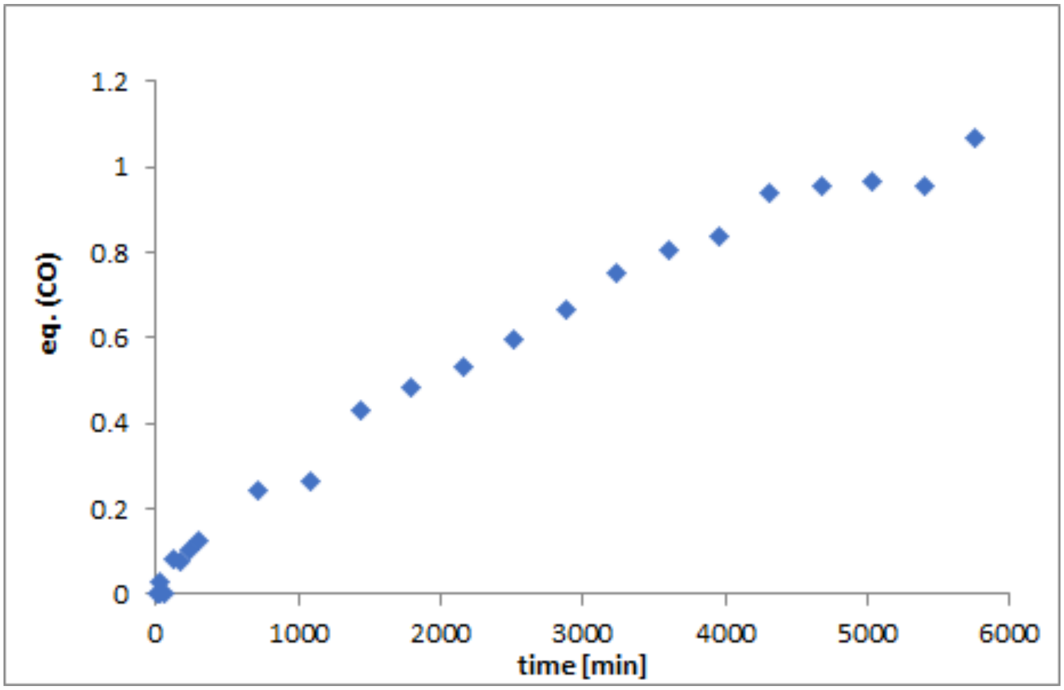

Experimental Part 2 (Biology)

Cell culture

The following human cell lines and their chemoresistant sublines were used in this study: control vector and FADD-dn-transfected Burkitt-like lymphoma (BJAB) cells,[4] which stably express a dominant-negative FADD mutant lacking the N-terminal death effector domain (kindly donated by Prof. Dr. P. T. Daniel, Charité Berlin); control vector and Bcl2-transfected melanoma cells (Mel-HO),[5] which stably overexpress the anti-apoptotic protein Bcl2 (kindly provided by Dr. Eberle, Charité Berlin); breast cancer cells MCF7, which are caspase-3 negative and their caspase-3 positive variant after transfection with caspase-3 (kindly provided by Dr. Jänicke, University of Düsseldorf); control vector and smac/DIABLO-transfected leukemic T-cells (Jurkat), which stably overexpress the pro-apoptotic protein smac/DIABLO (kindly donated by Prof. Dr. S. Fulda, University of Ulm);[6] leukemic B-cell precursor (Nalm-6) and its vincristine (Nalm/VCR) and daunorubicin (Nalm/DAUNO) resistant sublines. Both cell lines show an expression of p-glycoprotein with multidrug resistance. All cell lines were cultured in RPMI 1640 supplemented with 10% fetal calf serum, except for Mel-HO and MCF7 cells, which were cultured in DMEM with 10% fetal calf serum.

Primary cells

Primary leukocytes (PBMC) from a healthy person were obtained from peripheral blood donated by the co-author A. Prokop. Mononuclear cells were separated by centrifugation over a Ficoll gradient. After separation, the percentage of leukemic lymphoblasts or myelocytes exceeded 95%, in accordance with a former study.[7] Primary cells were immediately seeded in 6-well plates and exposed to the agents for the apoptosis assays.

Measurement of cell death by LDH release assay

Cytotoxicity was measured by release of lactate dehydrogenase (LDH) as described previously.[8] After incubation with different concentrations of complex **3** (NAHO27) for 2 h, LDH released by BJAB cells was measured in cell culture supernatants using a cytotoxicity detection kit from Boehringer Mannheim (Mannheim, Germany). The supernatants were centrifuged at 300 × g for 5 min. Cell-free supernatants (20 µl) were diluted with 80 µl PBS and 100 µl reaction mixture were added. Then, the time-dependent formation of the reaction product was quantified photometrically at 490 nm. The maximum amount of LDH activity released by the cells was determined by lysis of the cells using 0,1% Triton X-100 in culture medium and was set as 100% cell death.

Determination of cell concentration and cell viability

Cell viability was determined by CASY® Cell Counter + Analyzer System of Schaerfe System GmbH (Reutlingen, Germany). Settings were specifically defined for the requirements of the cells used. With this system the cell concentration is analyzed simultaneously in three different size ranges: cell debris, dead cells, and viable cells were determined in one measurement.[9] BJAB cells were seeded at a density of 1×10^5 cells/ml and treated with different concentrations of NAHO27 (**3**), non-treated cells served as controls. After 24 h of incubation, cells were resuspended properly and 100 µl of each well were diluted in 10 ml CASYton (ready-to-use isotonic saline solution) for an immediate automated count of the cells.

Measurement of DNA fragmentation

Apoptotic cell death was determined by a modified cell cycle analysis,[10] which detects DNA fragmentation at the single cell level. For measurement of DNA fragmentation cells were seeded at a density of 1 × 105 cells/ml and treated with different concentrations of NAHO27 (**3**). After 72 h of incubation, cells were collected by centrifugation at 300 × g for 5 min, washed with PBS at 4 °C and fixed in PBS/2% (v/v) formaldehyde on ice for 30 min. After fixation, cells were incubated with ethanol / PBS (2:1, v/v) for 15 min, pelleted and resuspended in PBS containing 40 µg/ml RNase A. After incubation for 30 min at 37 °C, cells were pelleted again and finally resuspended in PBS containing 50 µg/ml propidium iodide. Nuclear DNA fragmentation was then quantified by flow cytometric determination of hypodiploid DNA. Data were collected and analyzed using a FACScan (Becton Dickinson, Heidelberg, Germany) equipped with the CELLQuest software. Data are given in % hypoploidy (subG1), which reflects the number of apoptotic cells.

Measurement of the mitochondrial permeability transition After incubation with different concentrations of NAHO27 (**3**), BJAB cells were collected by centrifugation at 300 × g at 4 °C for 5 min. Mitochondrial permeability transition was then determined by staining the cells with 5,5,6,6-tetrachloro-1,1,3,3-tetraethyl-benzimidazolylcarbocyanin iodide (JC-1; Molecular Probes, Leiden, The Netherlands) as described.[11,12] Cells (1 × 105) were resuspended in 500 µl phenol red-

free RPMI 1640 without supplements and JC-1 was added to give a final concentration of 2,5 µg/ml. The cells were incubated for 30 min at 37 °C and moderate shaking. Control cells were likewise incubated in the absence of JC-1 dye. The cells were harvested by centrifugation at 1500 rpm, 4 °C for 5 min, washed with ice-cold PBS and resuspended in 200 µl PBS at 4 °C. Mitochondrial permeability transition was then quantified by flow cytometric determination of cells with decreased fluorescence, i.e. with mitochondria displaying a lower membrane potential ($\Delta\Psi_m$). Data were collected and analyzed using a FACScan (Becton Dickinson) equipped with the CELL Quest software. Data are given in % cells with low $\Delta\Psi_m$, which reflects the number of cells undergoing mitochondrial apoptosis.

Annexin V-propidiumiodid binding assay

Early and late apoptotic rates were assessed with flow cytometry using the annexin V–fluorescein isothiocyanate/propidium iodide (PI) kit (BD Pharmingen, San Diego, CA, USA), in which annexin V binds to exposed phosphatidylserine of the early apoptotic cells, [13,14] whereas PI stained the cells that had an increased membrane permeability. Samples were prepared according to the manufacturer's instructions. Flow cytometry analysis was performed after 48h using a FACS-Calibur cytometer (Becton Dickinson, San Jose, CA). Cells, which were Annexin-V-FITC-negative and PI-negative (Annexin-V-/PI-) were classified as vital, while Annexin-V-FITC-negative and PI-positive cells (Annexin-V-/PI+) were classified as necrotic. PI-positive and Annexin-V-FITC-positive cells (Annexin-V+/PI+) were classified as late apoptotic, and Annexin-V-FITC-positive but PI-negative (Annexin-V+/PI-) cells were classified as early apoptotic.

Western blot analysis

After incubation for 24 with **6**, **8**, **10** µM of NAHO27 (**3**), BJAB cells were washed twice with PBS and lysed in buffer containing 10 mM Tris/HCl, pH 7.5, 300 mM NaCl, 1% Triton X-100, 2 mM $MgCl_2$, 5 µM ethylenediaminetetraacetic acid (EDTA) and one protease inhibitor cocktail tablet (Roche Diagnostics, Germany). Protein concentration was determined using the bicinchoninic acid assay[15] from Pierce (Rockford, IL, USA) and equal amounts of protein (usually 40 µg per lane) were separated by SDS-PAGE.[16] Then, immunoblotting of protein onto nitrocellulose membranes (Schleicher and Schuell, Dassel, Germany) was performed exactly as described.[17] After blotting, the membrane was blocked for 1 h in PBST (PBS, 0.05% Tween-20) containing 5% non-fat dry milk and incubated with primary antibody for 1 h. After the membrane had been washed three times in PBST, secondary antibody in PBST was applied for 1 h. Finally, the membrane was washed in PBST again and protein bands were visualized using the ECL enhanced chemiluminescence system (Amersham Buchler, Braunschweig, Germany).

Gene expression analysis

For the analysis of the differential expression of multiple genes involved in the different apoptosis pathways, we used apoptosis-specific RT2 profiler (polymerase chain reaction) PCR expression arrays (SuperArray PAHS-012; SABiosciences Corporation, Frederick, MD, USA), according to the manufacturer's instructions. Total RNA was extracted from BJAB cells incubated with NAHO27 (**3**) (10 µM) for 15 h, and RNAs were treated with DNase I (2 U/µl) to eliminate possible genomic DNA contamination. Total RNA (700 ng/µl) was then used as a template for the synthesis of a cDNA probe and subjected to quantitative real-time PCR SuperArray analysis according to the manufacturer's instructions using a LightCycler480 (Roche Diagnostics). Results were analyzed using SuperArray Analyzer Software, and the data are given as fold change of expression of the respective genes when compared with control cells incubated in vehicle-containing medium for 15 h.

References

1 J. Panteleev, R. Y. Huang, E. K. J. Lui and M. Lautens, *Org. Lett.*, 2011, **13**, 5314-5317.
2 L. Gehringer, C. Bourgogne, D. Guillon and B. Donnio, *J. Am. Chem. Soc.*, 2004, **126**, 3856-3867.
3 J. L. Jeffrey and R. Sarpong, *Tetrahedron Lett.*, 2009, **50**, 1969-1972.
4 T. Wieder, F. Essmann, A. Prokop, K. Schmelz, K. Schulze-Osthoff, R. Beyaert, B. Dörken and P. T. Daniel, *Blood,* 2001, **97**, 1378-1387.
5 P. Jesse, G. Mottke, J. Eberle, G. Seifert, G. Henze and A. Prokop. *Pediatr. Blood Cancer,* 2009, **52**, 464-469.
6 M. Vogler, S. Giagkousiklidis, F. Genze, J. E, Gschwend, K. M. Debatin and S. Fulda, *Oncogene*, 2005, **24**, 7190-7202.
7 A. Prokop, T. Wieder, I. Sturm, F. Essmann, K. Seeger, C. Wuchter, W. D. Ludwig, G. Henze, B. Dörken and P. T. Daniel, *Leukemia,* 2000, **14**, 1606-1613.
8 T. Wieder, C. E. Orfanos and C. C Geilen, *J. Biol. Chem.*, 1998, **273**, 11025-11031.
9 R. Voisard, P. C. Dartsch, U. Seitzer, D. Roth, M. Kochs and V. Hombach, *Vasa Suppl.*, 1991, **33**, 140-141.
10 F. Essmann, T. Wieder, A. Otto, E. C. Muller, B. Dorken and P. T. Daniel, *Biochem. J.*, 2000, **346**, 777-783.
11 M. Reers, S. T. Smiley, C. Mottola-Hartshorn, A. Chen, M. Lin and L. B. Chen, *Methods Enzymol.,* 1995, **260**, 406-417.

12 I. H. Lambert, E. K. Hoffmann and F. Jorgensen, *J. Membr. Biol.,* 1989, **111**, 113-131.
13 V. A. Fadok, D. Xue and P. Henson, *Cell Death Differ*., 2001, **8**, 582-587
14 R. A. Schlegel and P. Williamson, *Cell Death Differ.,* 2001, **8**, 551-563
15 P. K. Smith, R. I. Krohn, G. T. Hermanson A. K. Mallia, F. H. Gartner, M. D. Provenzano, E. K. Fujimoto, N. M. Goeke, B. J. Olson and D. C. Klenk, *Anal. Biochem.*, 1985, **150**, 76-85.
16 U. K. Laemmli, *Nature*, 1970, **227**, 680-685
17 T. Wieder, C. C. Geilen, M. Wieprecht, A. Becker and C. E. Orfanos, *FEBS Lett*., 1994, **345**, 207-210.
